# Atypical Mixed Variant of Pericallosal Lipoma With Splenial Dysgenesis

**DOI:** 10.7759/cureus.11532

**Published:** 2020-11-17

**Authors:** Mohammed Al-Hameed, Kaitlin M Zaki-Metias, Fadi Deeb, Kamran A Shah

**Affiliations:** 1 Department of Radiology, St. Joseph Mercy Oakland Hospital, Pontiac, USA; 2 Department of Radiology, Ross University School of Medicine, Bridgetown, BRB; 3 Neuroradiology, Huron Valley Radiology, Ypsilanti, USA

**Keywords:** pericallosal lipoma, intracranial lipoma, mixed variant lipoma, tubulonodular, curvilinear

## Abstract

Pericallosal lipomas are rare benign intracranial masses that arise during embryonic development, typically categorized into tubulonodular and curvilinear subtypes. A mixed variant of both tubulonodular and curvilinear subtypes is very rare. Patients with pericallosal lipomas may be asymptomatic or may have different presentations, such as headaches. Conservative medical management is the mainstay of therapy for those without epileptic seizures or associated vascular malformations. We present a case of a mixed variant pericallosal lipoma in a patient with chronic headaches that were diagnosed using head computed tomography (CT) and brain magnetic resonance imaging (MRI).

## Introduction

Intracranial lipomas are rare congenital malformations caused by the overproliferation of lipocytes within the embryological leptomeninx [1]. Pericallosal lipomas account for up to 65% of all intracranial lipomas. Although they are often incidental prenatal findings, patients may present later in life with epilepsy, headache, psychomotor retardation, or other neurological symptoms [2].

They can be classified into two subgroups based on morphology: tubulonodular and curvilinear. The tubulonodular type is the most common form, characterized by a bulky tubular mass along the anterior corpus callosum and typically measures greater than 2 cm in axial diameter. On the other hand, the curvilinear type is a thinner ribbon-like mass, curving around the splenium of the corpus callosum, and usually measures less than 1 cm in axial diameter. A mixed variant of both types is exceedingly rare, with very few cases reported in the literature [3-6]. 

Tubulonodular lipomas are formed at an earlier stage of development; they have a higher incidence of corpus callosum dysgenesis and can be associated with calcifications, frontal bone defects, and cortical dysplasia [2, 6]. Pericallosal lipomas can also be associated with rare disorders such as Pai syndrome, in which cleft palate, cleft lip, and cutaneous facial polyps may be seen, as well as pericallosal lipomas [7].

Differential diagnoses include intracranial aneurysm, dural venous sinus thrombosis, pericallosal hemorrhage, dermoid cysts, and other intracranial tumors [8, 9].

Prenatal sonographic examination typically demonstrates a hyperechoic midline mass, suggestive of pericallosal lipoma. However, postnatally, the diagnosis can be established with CT and MRI [2, 8]. On CT, pericallosal lipomas show a well-demarcated area of fat density, between -40 to -100 Hounsfield units, that does not enhance after intravenous contrast administration. Peripheral calcifications may be present [9]. On MRI, pericallosal lipomas do not enhance after administration of gadolinium-based contrast agents. They are typically hyperintense on both T1 and T2-weighted images. This hyperintensity is homogenously suppressed on fat suppression sequences [2, 10].

## Case presentation

A 65-year-old female with no significant past medical history presents with chronic headaches for many years. She states her headaches are global and do not localize to any one region. She reports mild intermittent nausea but denies vomiting, aura, photophobia, or phonophobia. She denies any visual disturbances or other neurological symptoms. She has no known personal or family history of malignancy, aneurysm, or epilepsy. She denies recent head trauma. She takes no medications. Vital signs are within the normal range. Physical examination is unremarkable, with no focal neurological deficits noted. Basic lab results are within normal limit.

**Figure 1:**
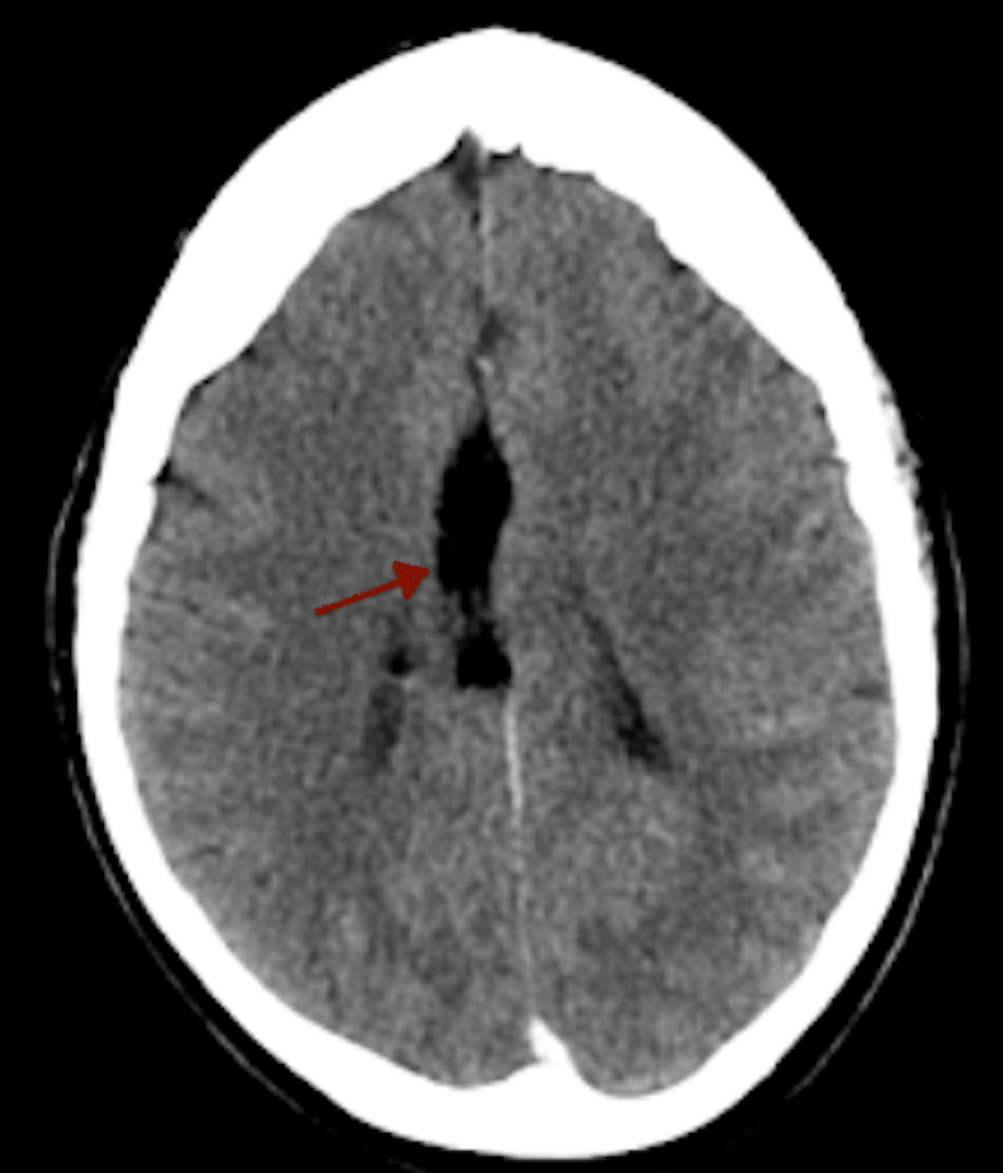
Axial non-contrast CT of the head Axial non-contrast CT of the head demonstrates a midline hypodense fat-attenuating lesion (red arrow) measuring up to 1.5 cm in axial diameter.

**Figure 2:**
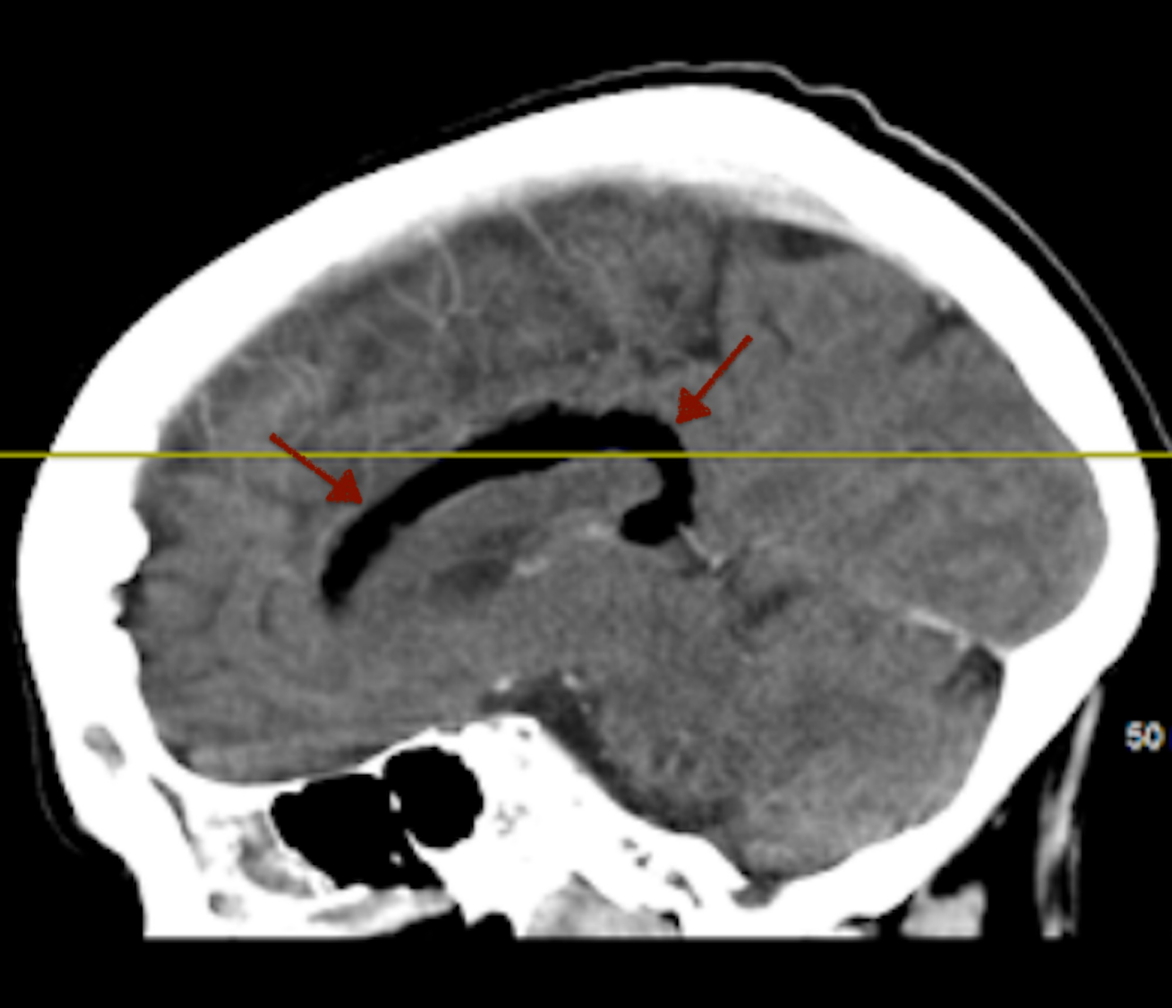
Sagittal CT of the head Sagittal CT of the head demonstrates a tubular area of hypodense fat attenuation (red arrows) along the superior callosal margin that curves around the splenium of the corpus callosum.

**Figure 3:**
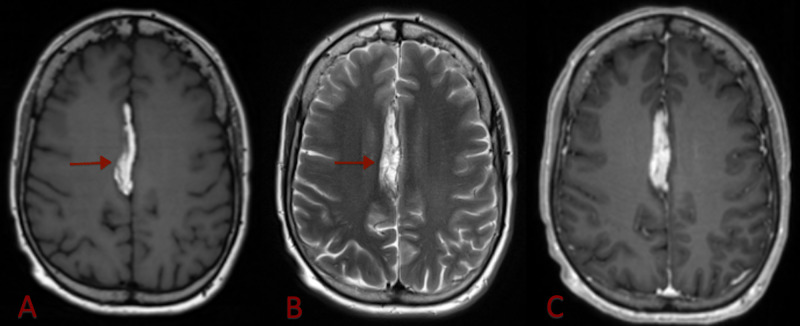
Axial magnetic resonance images of the brain Axial magnetic resonance images of the brain show a midline hyperintense lesion (red arrow) on both T1 pre-contrast (A) and T2 (B) weighted images. There is a lack of enhancement on T1 post-contrast image (C). These findings are consistent with fat.

**Figure 4:**
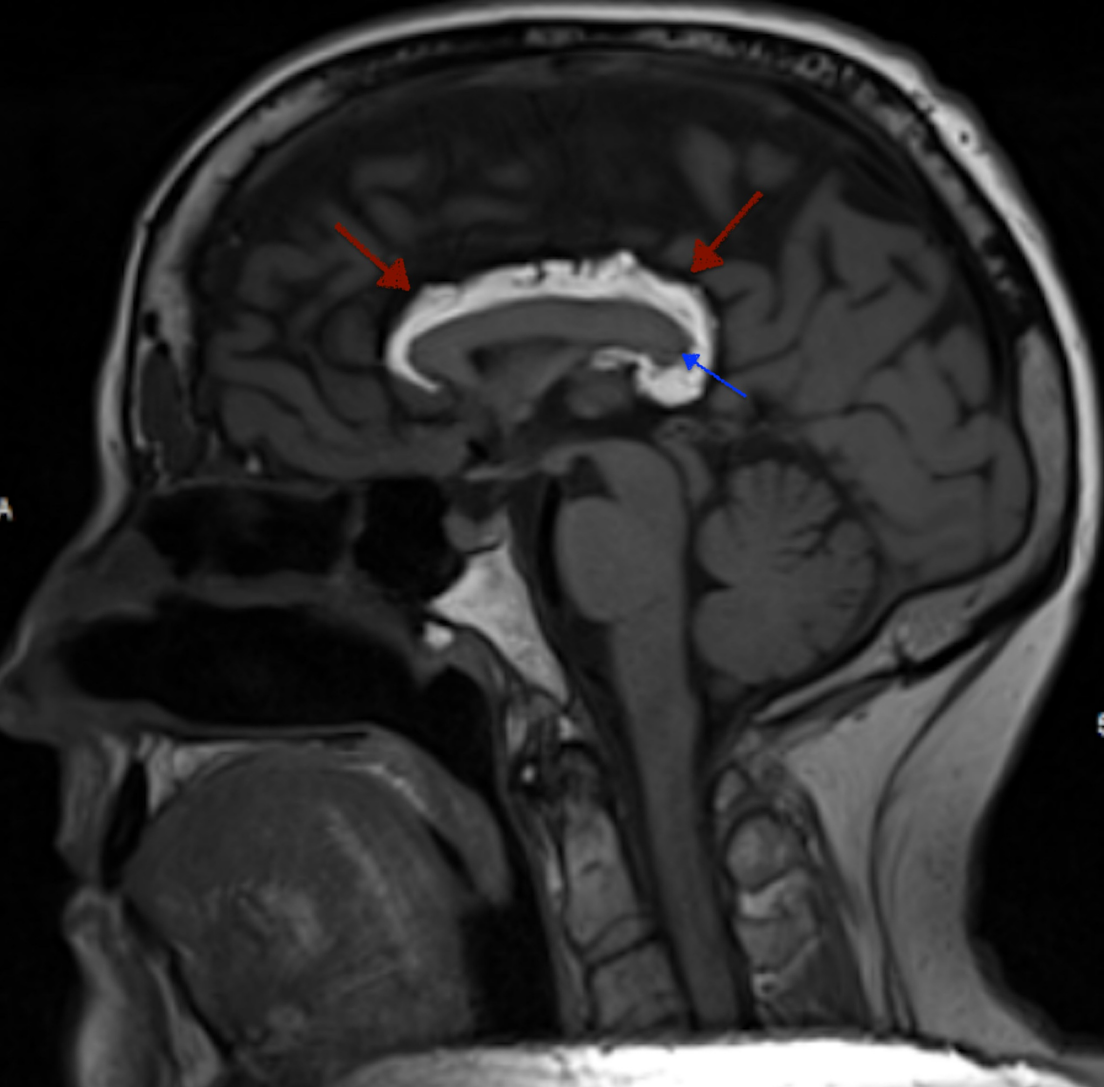
Sagittal pre-contrast T1-weighted MRI of the brain Sagittal pre-contrast T1-weighted MRI of the brain demonstrates a hyperintense lesion (red arrows) along the anterior and posterior aspects of the superior margin of the corpus callosum, curving around the splenium. Additionally, there is associated splenial hypoplasia (blue arrow) of the corpus callosum.

Non-contrast CT of the head (Figures 1-2) was performed and demonstrated a midline tubular area of hypodense fat attenuation along the entire length of the superior callosal margin, curving around the splenium. MRI of the brain with and without intravenous contrast (Figures 3-4) was subsequently obtained and redemonstrated a midline non-enhancing hyperintense tubular lesion on both T1, and T2 weighted images. The lesion extends along the anterior and posterior aspects of the superior margin of the corpus callosum and curves around the splenium. The lesion measures up to 1.5 cm in axial thickness and is additionally associated with splenial hypoplasia of the corpus callosum. These radiological findings are consistent with benign pericallosal lipoma.

After the diagnosis of pericallosal lipoma was made, the patient was reassured and managed conservatively.

## Discussion

Intracranial lipomas are rare and often an incidental finding [1, 9]. There are two subtypes of pericallosal lipomas, tubulonodular and curvilinear, which can be classified based on morphology. When the appearance of the lipoma does not correspond to only one subtype and involves both the anterior and the posterior portion of the corpus callosum, as in our case, this can make it a mixed variant instead [3, 4]. 

Lipomas are slowly progressive lesions; therefore, the prognosis is generally favorable. Medical therapy and conservative management are nearly always preferred for symptomatic lesions. However, there are two situations in which surgical intervention may be considered; in epilepsy and with vascular lesions [11].

Epileptic seizures, a common symptomatic finding, are treated medically, with surgery often reserved for refractory cases. Recent studies indicate that the lipomas themselves may not be the cause of epilepsy, further discounting the benefits of surgical intervention [12]. Lipomas associated with unstable vascular lesions, such as aneurysms or arteriovenous malformations, require careful attention during resection due to the tendency of these lesions to adhere to the surrounding neural architecture. Evidence on surgical intervention concerning other medically refractory symptoms, such as vertigo or headache, remains inconclusive; however, these symptoms are primarily treated symptomatically [11]. 

## Conclusions

Pericallosal lipomas, in general, are rare benign masses that arise during embryonic development. They can be associated with variable degrees of corpus callosum dysgenesis. Head CT and brain MRI with and without intravenous contrast are needed for the diagnosis. A mixed variant of both tubulonodular and curvilinear subtypes is very rare. Conservative medical management is the mainstay of therapy for those without epileptic seizures or associated vascular malformations, where surgical intervention may be considered. Appreciating the variable presentations and the different radiological morphologies can provide a better understanding of pericallosal lipomas and aid in promoting the diagnosis and the treatment plan.
